# The distribution of the ratio of products of independent zero mean normal random variables

**DOI:** 10.1007/s00184-026-01045-2

**Published:** 2026-08-24

**Authors:** Robert E. Gaunt, Heather L. Sutcliffe

**Affiliations:** Department of Mathematics, The University of Manchester, Oxford Road, Manchester, M13 9PL UK

**Keywords:** Normal distribution, Ratios of random variables, Products of random variables, Meijer *G*-function, Primary 60E05, 62E15, Secondary 33C60, 41A60

## Abstract

Let $X_1,\ldots ,X_M$ and $Y_1,\ldots ,Y_N$ be independent zero mean normal random variables with variances $\sigma _{X_i}^2$, i=1,…,M, and $\sigma _{Y_j}^2$, j=1,…,N, respectively, and let $X=X_1\cdots X_M$ and $Y=Y_1\cdots Y_N$. In this paper, we derive the exact probability density function of the ratio *X*/*Y*. We apply this formula to derive exact formulas for the cumulative distribution function and the characteristic function. We also obtain further distributional properties, including asymptotic approximations for the probability density function, tail probabilities and the quantile function.

## Introduction

Ratios and products of random variables arise in applications throughout probability and statistics, as well as the biological and physical sciences; for some examples of application areas see Nadarajah and Dey (2006). As such, there is now an extensive literature concerning the distributional theory of ratios and products of random variables, with exact formulas derived for many classical probability distributions; see Nadarajah (2007) for an overview of some of this literature.

In this paper, we study the exact distribution of the ratio of products of independent zero mean normal random variables, and in doing so fill a clear gap in the literature. More specifically, let $X_1,\ldots ,X_M$ and $Y_1,\ldots ,Y_N$ be independent normal random variables with zero mean and variances $\sigma _{X_i}^2$, i=1,…,M, and $\sigma _{Y_j}^2$, j=1,…,N, respectively. Also, let $\sigma _{M}=\sigma _{X_1}\cdots \sigma _{X_M}$ and $s_N=\sigma _{Y_1}\cdots \sigma _{Y_N}$. We use the convention that the empty product is set to one. We thus set $\sigma _0=1$ and $s_0=1$, and set X=1 when M=0, and Y=1 when N=0. In this paper, we study the distribution of the ratio Z=X/Y, deriving a number of its key distributional properties, including the probability density function (PDF), cumulative distribution function (CDF), characteristic function, fractional moments, and also derive asymptotic approximations for the PDF, tail probabilities and the quantile function. These results are stated and proved in Sect. 2.

The general formulas for the PDF, CDF and characteristic function of Sect. 2 are stated in terms of the Meijer *G*-function (defined in Appendix A). In Sect. 3, we present some special cases in which these formulas take a simpler form. We now briefly review some special cases that have already been treated in the literature. A closed-form formula for the PDF of the product $X=X_1\cdots X_M$, expressed in terms of the Meijer *G*-function, was obtained by Springer and Thompson (1970):

1.1 $$\begin{aligned} f_X(x)=\frac{1}{(2\pi )^{M/2}\sigma _M}G^{M,0}_{0,M}\bigg (\frac{x^2}{2^M\sigma ^2_M} \; \bigg | \;{\begin{array}{c} - \\ \\ 0,\ldots ,0 \end{array}}\bigg ), \quad x\in \mathbb {R}. \end{aligned}$$

In the case M=2, the PDF (1.1) for the product $X=X_1X_2$ takes the simpler form $f_X(x)=(\pi \sigma _2)^{-1}K_0(|x|/\sigma _2)$, $x\in \mathbb {R}$, where $K_0(x)$ is a modified Bessel function of the second kind (see Wishart and Bartlett 1932). However, further simplifications are unavailable for M≥3. It is well-known that the ratio of independent zero-mean normal random variables follows the Cauchy distribution, and so in the case M=N, the ratio Z=X/Y is distributed as the product of *M* independent Cauchy random variables. The PDF of the product of two independent Cauchy random variables was derived by Ride (1965), and more generally an expression for the PDF of the product of independent Cauchy random variables was obtained by Springer and Thompson (1966). Exact formulas for the PDF and CDF of *M* independent Cauchy random variables also follow as special cases of the formulas of Nadarajah (2012) for the product of *M* independent Student’s *t* distributed random variables, which are expressed in terms of the Meijer *G*-function.

The results of this paper may prove to be useful in application areas in which ratios of products of independent zero mean normal random variables arise. An example of a concrete setting in which this distribution arises is the analysis of path weights at the random initialization of a neural network. Under the initialization scheme of He et al. (2015), the individual weights are independent zero mean normal random variables, while the signed weight associated with a path is the product of the weights on its edges (Neyshabur et al. 2015). Thus, for two edge-disjoint paths containing *M* and *N* edges, respectively, the ratio of their signed path weights $X_1,\ldots ,X_M$ and $Y_1,\ldots ,Y_N$ is given by Z=X/Y, where $X=X_1\cdots X_M$ and $Y=Y_1\cdots Y_N$. Exact probabilities for the ratio *Z* can then be computed using our exact formulas without the need for simulations. Moreover, if one is interested in quantification of extreme imbalance between multiplicative paths, in which, for example the first path weight exceeds that of the second by a large factor *c*, then the probability $\mathbb {P}(|Z|>c)$ can be rapidly estimated using the asymptotic approximations of Theorem 2.7 without the need for simulation.

## The normal product ratio distribution

### Probability density function

In the following theorem, we provide a closed-form formula for the PDF $f_Z(z)$ of the ratio Z=X/Y which is expressed in terms of the Meijer *G*-function.

#### Theorem 2.1

Suppose M,N≥0 and M+N≥1. Let $X_1,\ldots ,X_M$ and $Y_1,\ldots ,Y_N$ be independent random variables where $X_i\sim N(0,\sigma ^2_{X_i})$, i=1,…,M, and $Y_j\sim N(0,\sigma ^2_{Y_j})$, j=1,…,N. Let $X=X_1\cdots X_M$ and $Y=Y_1\cdots Y_N$, and denote Z=X/Y. Then, for $z\in \mathbb {R}$,

2.2 $$\begin{aligned} f_Z(z)=\frac{2^{(N-M)/2}s_N}{\pi ^{(M+N)/2}\sigma _M}G^{M,N}_{N,M}\bigg (\frac{2^Ns_N^2}{2^M\sigma _M^2}z^2 \; \bigg | \;{\begin{array}{c} 0,\ldots ,0 \\ \\ 0,\ldots ,0 \end{array}}\bigg ). \end{aligned}$$

#### Proof

When M≥1 and N≥1 we have, for $z\in \mathbb {R}$,

$$\begin{aligned} f_Z(z)&=\int _{-\infty }^{\infty }|x|f_X(zx)f_Y(x)\,\textrm{d}x=2\int _{0}^{\infty }xf_X(zx)f_Y(x)\,\textrm{d}x\\&=\frac{2}{(2\pi )^{(M+N)/2}\sigma _Ms_N}\int ^{\infty }_{0}x\,G^{M,0}_{0,M}\bigg (\frac{z^2x^2}{2^M\sigma _M^2} \; \bigg | \;\begin{array}{c} - \\ \\ 0,\ldots ,0 \end{array}\bigg )G^{N,0}_{0,N}\bigg (\frac{x^2}{2^Ns_N^2} \; \bigg | \;{\begin{array}{c} -\\ \\ 0,\ldots ,0 \end{array}}\bigg )\,\textrm{d}x\\&=\frac{1}{(2\pi )^{(M+N)/2}\sigma _Ms_N}\int ^{\infty }_{0}G^{M,0}_{0,M}\bigg (\frac{z^2u}{2^M\sigma _M^2} \; \bigg | \;{\begin{array}{c} -\\ \\ 0,\ldots ,0 \end{array}}\bigg )G^{N,0}_{0,N}\bigg (\frac{u}{2^Ns_N^2} \; \bigg | \;\begin{array}{c} - \\ \\ 0,\ldots ,0 \end{array}\bigg )\,\textrm{d}u, \end{aligned}$$

where in the third step we used the PDF formula (1.1). On using formula 5.6.2(1) of Luke (1969) to evaluate the integral we obtain (2.2). When N=0 our PDF formula (2.2) reduces to the PDF (1.1) for $X=X_1\cdots X_M$, as given in Springer and Thompson (1970), and so the formula is also valid for M≥1 and N=0. Finally, we verify that formula (2.2) is also valid for M=0 and N≥1. In this case, by the standard formula for the PDF of a one-to-one transformation of a random variable, $f_Z(z)=f_{1/Y}(z)=z^{-2}f_Y(1/z)$, $z\in \mathbb {R}$, so that, by formula (1.1),

$$\begin{aligned} f_Z(z)&=\frac{1}{(2\pi )^{N/2}s_N}\frac{1}{z^2}G^{N,0}_{0,N}\bigg (\frac{1}{2^Ns^2_Nz^2} \; \bigg | \;\begin{array}{c} - \\ \\ 0,\ldots ,0 \end{array}\bigg ) =\frac{2^{N/2}s_N}{\pi ^{N/2}}G^{0,N}_{N,0}\bigg (2^Ns_N^2z^2 \; \bigg | \;{\begin{array}{c} 0,\ldots ,0 \\ \\ - \end{array}}\bigg ), \end{aligned}$$

where the second equality follows from applying the identities (A.30) and (A.31). This confirms that formula (2.2) holds for M=0 and N≥1. □

### Cumulative distribution function

Let $F_Z(z)=\mathbb {P}(Z\le z)$ denote the CDF of Z=X/Y. In the following theorem, we provide closed-form formulas for the CDF of *Z*.

#### Theorem 2.2

Let M,N≥0 and M+N≥1. Then, for $z\in \mathbb {R}$,

2.3 $$\begin{aligned} F_Z(z)&=\frac{1}{2}+\frac{2^{(N-M-2)/2}s_N}{\pi ^{(M+N)/2}\sigma _M}z\,G^{M,N+1}_{N+1,M+1}\bigg (\frac{2^Ns_N^2}{2^M\sigma _M^2}z^2 \; \bigg | \;{\begin{array}{c} \frac{1}{2},0,\ldots ,0 \\ \\ 0,\ldots ,0,-\frac{1}{2} \end{array}}\bigg ) \end{aligned}$$

2.4 $$\begin{aligned}&=\frac{1}{2}+\frac{\textrm{sgn}(z)}{2\pi ^{(N+M)/2}}G^{M,N+1}_{N+1,M+1}\bigg (\frac{2^Ns_N^2}{2^M\sigma _M^2}z^2 \; \bigg | \;{\begin{array}{c} 1,\frac{1}{2},\ldots ,\frac{1}{2}\\ \\ \frac{1}{2},\ldots ,\frac{1}{2},0 \end{array}}\bigg ), \end{aligned}$$

where $\textrm{sgn}(z)$ is the sign function, $\textrm{sgn}(z)=-1$ for z<0, $\textrm{sgn}(0)=0$, $\textrm{sgn}(z)=1$ for z>0.

#### Proof

As the PDF (2.2) is symmetric about the origin, we have that, for z>0,

$$\begin{aligned} F_Z(z)&=\frac{1}{2}+\frac{2^{(N-M)/2}s_N}{\pi ^{(M+N)/2}\sigma _M}\int _{0}^{z}G^{M,N}_{N,M}\bigg (\frac{2^Ns_N^2}{2^M\sigma _M^2}x^2 \; \bigg | \;\begin{array}{c} 0,\ldots ,0 \\ \\ 0,\ldots ,0 \end{array}\bigg )\,\textrm{d}x\\&=\frac{1}{2}+\frac{1}{2\pi ^{(M+N)/2}}\int _{0}^{\frac{2^Ns_N^2}{2^M\sigma _M^2}z^2}u^{-1/2}G^{M,N}_{N,M}\bigg (u \; \bigg | \;\begin{array}{c} 0,\ldots ,0 \\ \\ 0,\ldots ,0 \end{array}\bigg )\,\textrm{d}u\\&=\frac{1}{2}+\frac{1}{2\pi ^{(M+N)/2}}\bigg [u^{1/2}G^{M,N+1}_{N+1,M+1}\bigg (u \; \bigg | \;{\begin{array}{c} \frac{1}{2},0,\ldots ,0 \\ \\ 0,\ldots ,0,-\frac{1}{2} \end{array}}\bigg )\bigg ]^{\frac{2^Ns_N^2}{2^M\sigma _M^2}z^2}_{0} \\&=\frac{1}{2}+\frac{2^{(N-M-2)/2}s_N}{\pi ^{(M+N)/2}\sigma _M}\,z\,G^{M,N+1}_{N+1,M+1}\bigg ( \frac{2^Ns_N^2}{2^M\sigma _M^2}z^2\; \bigg | \;{\begin{array}{c} \frac{1}{2},0,\ldots ,0 \\ \\ 0,\ldots ,0,-\frac{1}{2} \end{array}}\bigg ), \end{aligned}$$

where the integral was evaluated using (A.32). We also used that the *G*-function in the third equality evaluated at u=0 is equal to zero, which can be seen from the contour integral representation (A.28) of the *G*-function. This confirms that formula (2.3) holds for z>0; the verification for z<0 is similar and is hence omitted. Lastly, formula (2.4) follows from (2.3) by an application of the identity (A.30), in which the cases z>0 and z<0 are treated separately. □

#### Remark 2.3

When specialised to the case N=0, the formulas (2.3) and (2.4) represent new formulas for the CDF of the product $X=X_1\cdots X_M$. An alternative formula for the CDF of *X*, also expressed in terms of the Meijer *G*-function, is given in (Stojanac et al. 2017, Lemma 2.1).

### Characteristic function

In the following theorem, we provide a closed-form formula for the characteristic function of *Z*, which we denote by $\varphi _Z(t)=\mathbb {E}[\textrm{e}^{\textrm{i}tZ}]$.

#### Theorem 2.4

(i) Suppose M≥1 and N≥0. Then, for $t\in \mathbb {R}$,

2.5 $$\begin{aligned} \varphi _Z(t) =\frac{1}{\pi ^{(N+M-1)/2}}G^{N+1,M-1}_{M-1,N+1}\bigg (\frac{2^M\sigma _M^2t^2}{2^{N+2}s_N^2} \; \bigg | \;{\begin{array}{c} \frac{1}{2},\ldots ,\frac{1}{2} \\ \\ 0,\frac{1}{2},\ldots ,\frac{1}{2} \end{array}}\bigg ). \end{aligned}$$

(ii) Suppose M=0 and N≥1. Then, for $t\in \mathbb {R}$,

2.6 $$\begin{aligned} \varphi _Z(t) =\frac{1}{\pi ^{(N-1)/2}}G^{N+1,0}_{0,N+2}\bigg (\frac{t^2}{2^{N+2}s_N^2} \; \bigg | \;{\begin{array}{c} - \\ \\ 0,\frac{1}{2},\ldots ,\frac{1}{2} \end{array}}\bigg ). \end{aligned}$$

#### Proof

(i) Suppose M≥1 and N≥0. Since the PDF (2.2) is symmetric about the origin we have that, for $t\in \mathbb {R}$,

2.7 $$\begin{aligned} \varphi _Z(t)=\mathbb {E}[\cos (tZ)]&= \frac{2^{(N-M+2)/2}s_N}{\pi ^{(N+M)/2}\sigma _M}\int _{0}^{\infty }\cos (tz)G^{M,N}_{N,M}\bigg (\frac{2^Ns_N^2}{2^M\sigma _M^2}z^2 \; \bigg | \;\begin{array}{c} 0,\ldots ,0 \\ \\ 0,\ldots ,0 \end{array}\bigg )\,\textrm{d}z \nonumber \\&=\frac{2^{(N-M+2)/2}s_N}{\pi ^{(N+M-1)/2}\sigma _M|t|}G^{M,N+1}_{N+2,M}\bigg (\frac{2^{N+2}s_N^2}{2^M\sigma _M^2}\frac{1}{t^2} \; \bigg | \;{\begin{array}{c} \frac{1}{2},0,\ldots ,0 \\ \\ 0,\ldots ,0 \end{array}}\bigg )\nonumber \\&=\frac{2^{(N-M+2)/2}s_N}{\pi ^{(N+M-1)/2}\sigma _M|t|}G^{M-1,N+1}_{N+1,M-1}\bigg (\frac{2^{N+2}s_N^2}{2^M\sigma _M^2}\frac{1}{t^2} \; \bigg | \;{\begin{array}{c} \frac{1}{2},0,\ldots ,0\\ \\ 0,\ldots ,0 \end{array}}\bigg )\nonumber \\&=\frac{1}{\pi ^{(N+M-1)/2}}G^{M-1,N+1}_{N+1,M-1}\bigg (\frac{2^{N+2}s_N^2}{2^M\sigma _M^2}\frac{1}{t^2} \; \bigg | \;{\begin{array}{c} 1,\frac{1}{2},\ldots ,\frac{1}{2} \\ \\ \frac{1}{2},\ldots ,\frac{1}{2} \end{array}}\bigg ), \end{aligned}$$

where the integral was evaluated using formula 5.6.3(18) of Luke (1969), the penultimate equality was obtained by using (A.29) and the final equality was obtained using (A.30). Finally, we obtain (2.5) from (2.7) by using (A.31).

(ii) The proof is similar to part (i), with the only difference being that we do not use identity (A.29) to reduce the *G*-function to one of lower order (we cannot do this here as M=0). □

#### Remark 2.5

As can be seen from the asymptotic behaviour of the PDF of *Z* as $|z|\rightarrow \infty$ (as described in Theorem 2.7 below), the moment generating function $M_Z(t)=\mathbb {E}[\textrm{e}^{tZ}]$ is not defined for N≥1. The moment generating function is only defined in the cases M=1, N=0 (for all $t\in \mathbb {R}$) and M=2, N=0 (for $|t|<1/\sigma _2$); this can be seen from the limiting form (2.14).

### Asymptotic behaviour of the distribution

In the following theorems, we present asymptotic approximations for the PDF, CDF and quantile function of the ratio Z=X/Y. We let $\overline{F}_Z(z)=\mathbb {P}(Z> z)$ denote the complementary CDF of *Z*. Since the distribution of *Z* is symmetric about the origin, $F_Z(-z)=\overline{F}_Z(z)$, so we only provide an asymptotic approximation for $\overline{F}_Z(z)$ as $z\rightarrow \infty$. Also, for 0<p<1, we denote the quantile function of *Z* by $Q(p)=F_Z^{-1}(p)$. Again, due to the symmetry of the distribution, Q(1-p)=-Q(p), and so we only provide asymptotic approximations for *Q*(*p*) as p→0. The proofs of the theorems are deferred until the end of this subsection.

#### Theorem 2.6

(i) Suppose M≥1, N≥0. Then, as z→0,

2.8 $$\begin{aligned} f_Z(z)\sim \frac{2^{(M+N-2)/2}s_N}{\pi ^{(M+N)/2}\sigma _M}\frac{(-\ln |z|)^{M-1}}{(M-1)!}. \end{aligned}$$

(ii) Suppose M=0, N≥1. Then, as z→0,

2.9 $$\begin{aligned} f_Z(z)\sim \frac{2^{(N-2)/2}}{\sqrt{N\pi }s_N^{1/N}}|z|^{-(N+1)/N}\exp \bigg (-\frac{N}{2(s_N|z|)^{2/N}} \bigg ). \end{aligned}$$

#### Theorem 2.7

Suppose M≥0, N≥1. Then, as $|z|\rightarrow \infty$,

2.10 $$\begin{aligned} f_Z(z)\sim \frac{2^{(M+N-2)/2}\sigma _M}{\pi ^{(N+M)/2}(N-1)!s_N}\frac{(\ln |z|)^{N-1}}{z^2}. \end{aligned}$$

Also, as $$z\rightarrow \infty $$,

2.11 $$\begin{aligned} \overline{F}_Z(z)\sim \frac{2^{(M+N-2)/2}\sigma _M}{\pi ^{(M+N)/2}(N-1)!s_N}\frac{(\ln (z))^{N-1}}{z}. \end{aligned}$$

#### Theorem 2.8

(i) Suppose M≥0 and N≥1. Then, as p→0,

2.12 $$\begin{aligned} Q(p)\sim -\frac{2^{(M+N-2)/2}\sigma _M}{\pi ^{(M+N)/2}s_N(N-1)!}\frac{\big (\ln (1/p)\big )^{N-1}}{p}. \end{aligned}$$

(ii) Suppose M≥1 and N=0. Then, as p→0,

2.13 $$\begin{aligned} Q(p)\sim -\bigg (\frac{2\sigma _M^{M/2}}{M}\ln \big (1/p\big )\bigg )^{M/2}. \end{aligned}$$

#### Remark 2.9

When M≥1, N=0, we have $Z=X=X_1\cdots X_M$. In this case, the following limiting forms for the PDF and tail probabilities were obtained by Gaunt (2017) and Leipus et al. (2023), respectively:

2.14 $$\begin{aligned} f_Z(z)&\sim \frac{2^{M/2-1}}{\sqrt{M\pi }\sigma _M^{1/M}}|z|^{1/M-1}\exp \bigg (-\frac{M}{2}\bigg (\frac{|z|}{\sigma _M}\bigg )^{2/M}\bigg ), \quad |z|\rightarrow \infty , \end{aligned}$$

2.15 $$\begin{aligned} \overline{F}_Z(z)&\sim \frac{2^{M/2-1}\sigma _M^{1/M}}{\sqrt{M\pi }}z^{-1/M}\exp \bigg (-\frac{M}{2}\bigg (\frac{z}{\sigma _M}\bigg )^{2/M}\bigg ), \quad z\rightarrow \infty . \end{aligned}$$

#### Remark 2.10

Part (i) of Theorem 2.6 tells us that, as z→0, the PDF $f_Z(z)$ is bounded for M=1,N≥0, but has a singularity as z→0 in the cases $M\ge 2, N\ge 0$ and M=0,N≥1. Since the PDF is bounded everywhere except for the singularity at the origin (for the values of *M* and *N* for which there is a singularity), the distribution of *Z* is unimodal with mode 0 when M≥1, N≥0. On the other hand, in the case M=0, N≥1, the limiting form (2.9) tells us that, $f_Z(z)\rightarrow 0$, as z→0, meaning that the distribution of *Z* is not unimodal (since the the PDF $f_Z(z)$ is symmetric about the origin).

It follows from the limiting form (2.10) that the mean of the ratio *Z* is not defined for M≥0, N≥1. In the case M=N=1, we recover the well-known fact that the mean of the Cauchy distribution is undefined. However, the fractional moments $\mathbb {E}[|Z|^r]$ exist for -1<r<1, as seen in the following proposition.

#### Proposition 2.11

Suppose M≥0, N≥0 with M+N≥1. Then, for -1<r<1,

2.16 $$\begin{aligned} \mathbb {E}[|Z|^r]=\frac{2^{(M-N)r/2}\sigma _M^r}{\pi ^{(M+N)/2}s_N^r}\bigg (\Gamma \Big (\frac{r+1}{2}\Big )\bigg )^M\bigg (\Gamma \Big (\frac{1-r}{2}\Big )\bigg )^N . \end{aligned}$$

When M≥1 and N=0, equation (2.16) holds for all r>-1, whilst when M=0 and N≥1, equation (2.16) holds for all r<1.

#### Proof

By independence, $\mathbb {E}[|Z|^r]=(\prod _{i=1}^M\mathbb {E}[|X_i|^r])(\prod _{j=1}^N\mathbb {E}[|Y_j|^{-r}])$, and the result now follows from the standard formula $\mathbb {E}[|V|^p]=2^{p/2}\sigma ^p \pi ^{-1/2}\Gamma ((p+1)/2)$ for p>-1, where $V\sim N(0,\sigma ^2)$. □

#### Proof of Theorem 2.6

(i) To simplify expressions, we let $x=2^{(N-M)/2}s_Nz/\sigma _M$. It suffices to consider the limit x↓0, since the PDF (2.2) is an even function in *z*. Using the contour integral definition of the Meijer-*G* function (A.28) followed by the residue theorem gives that

2.17 $$\begin{aligned} G^{M,N}_{N,M}\bigg (x^2 \; \bigg | \;\begin{array}{c} 0,\ldots ,0 \\ \\ 0,\ldots ,0 \end{array}\bigg )&=\frac{1}{2\pi \textrm{i}}\int _L\big (\Gamma (s)\big )^M\big (\Gamma (1-s)\big )^Nx^{-2s}\,\textrm{d}s\nonumber \\&=\sum _{s^*\in A}\textrm{Res}\big [\big (\Gamma (s)\big )^M\big (\Gamma (1-s)\big )^Nx^{-2s}, \,s=s^*\big ], \end{aligned}$$

where the contour *L* is a loop encircling the poles of the gamma functions and *A* is the collection of the poles of the gamma functions. We now compute the residues in (2.17), starting with the pole at s=0, which amounts to finding the coefficient of $s^{-1}$ in the Laurent expansion of $(\Gamma (s))^Mx^{-2s}$. A simple calculation using the expansions $\Gamma (s)=1/s+\gamma +o(1)$, as s→0, where γ is the Euler-Mascheroni constant, and $x^{-2\,s} =\sum _{i=0}^{\infty }(-2\ln (x))^is^i/{i!}$ reveals that

$$\begin{aligned} \textrm{Res}\big [\big (\Gamma (s)\big )^M\big (\Gamma (1-s)\big )^Nx^{-2s},\,s=0\big ]\sim \frac{(-2\ln (x))^{M-1}}{(M-1)!},\quad x\downarrow 0. \end{aligned}$$

For the other poles, the residue is readily seen to be of a smaller asymptotic order in the limit x↓0 than the residue at s=0. Hence, as x→0 (equivalently z→0),

2.18 $$\begin{aligned} G^{M,N}_{N,M}\bigg (x^2 \; \bigg | \;\begin{array}{c} 0,\ldots ,0 \\ \\ 0,\ldots ,0 \end{array}\bigg )&\sim \frac{(-2\ln |x|)^{M-1}}{(M-1)!}\sim \frac{(-2\ln |z|)^{M-1}}{(M-1)!}, \end{aligned}$$

from which we deduce the limiting form (2.8).

(ii) We now suppose M=0; in this case we set $x=2^{N/2}s_Nz$. Then by identity (A.31) and the limiting form (A.33) we obtain that

$$\begin{aligned} G^{0,N}_{N,0}\bigg (x^2 \; \bigg | \;{\begin{array}{c} 0,\ldots ,0 \\ \\ - \end{array}}\bigg )&=G^{N,0}_{0,N}\bigg (x^{-2} \; \bigg | \;{\begin{array}{c} - \\ \\ 1,\ldots ,1 \end{array}}\bigg )\sim \frac{(2\pi )^{(N-1)/2}}{\sqrt{N}}|x|^{-(N+1)/N}\exp \big (-N|x|^{-2/N}\big ), \end{aligned}$$

as x→0. The limiting form (2.9) now follows on setting $x=2^{N/2}s_Nz$. □

#### Proof of Theorem 2.7

(i) By applying identity (A.31) followed by (A.30) we have that

2.19 $$\begin{aligned} f_Z(z)&=\frac{2^{(N-M)/2}s_N}{\pi ^{(M+N)/2}\sigma _M}G^{M,N}_{N,M}\bigg (\frac{2^Ns_N^2}{2^M\sigma _M^2}z^2 \; \bigg | \;\begin{array}{c} 0,\ldots ,0 \\ \\ 0,\ldots ,0 \end{array}\bigg )\nonumber \\&=\frac{2^{(N-M)/2}s_N}{\pi ^{(M+N)/2}\sigma _M}G^{N,M}_{M,N}\bigg (\frac{2^M\sigma _M^2}{2^Ns_N^2}\frac{1}{z^2} \; \bigg | \;{\begin{array}{c} 1,\ldots ,1 \\ \\ 1,\ldots ,1 \end{array}}\bigg )\nonumber \\&=\frac{2^{(N-M)/2}s_N}{\pi ^{(M+N)/2}\sigma _M}u^2G^{N,M}_{M,N}\bigg (u^2 \; \bigg | \;\begin{array}{c} 0,\ldots ,0 \\ \\ 0,\ldots ,0 \end{array}\bigg ), \end{aligned}$$

where $u=2^{(M-N)/2}\sigma _M/(s_Nz)$. The limiting form (2.10) now follows from applying the limiting form (2.18) to (2.19).

(ii) By (2.10), we have that, as $z\rightarrow \infty$,

$$\begin{aligned} \overline{F}_Z(z)=\int _z^\infty f_Z(x)\,\textrm{d}x\sim \frac{2^{(M+N-2)/2}\sigma _M}{\pi ^{(M+N)/2}(N-1)!s_N}\int _{z}^{\infty }\frac{(\ln (x))^{N-1}}{x^2}\,\textrm{d}x, \end{aligned}$$

and since $\int _{z}^{\infty }(\ln (x))^{N-1}/{x^2}\,\textrm{d}x\sim (\ln (z))^{N-1}/z$, as $z\rightarrow \infty$, we obtain (2.11).

□

To prove Theorem 2.8 we use the following lemma which is a special case of (Gaunt and Ye 2025, Lemma 4.2).

#### Lemma 2.12

Let a,A,z>0 and $m\in \mathbb {R}$. Let $g:(0,\infty )\rightarrow \mathbb {R}$ be a function such that g(x)→0 as $x\rightarrow \infty$. Consider the solution *x* of the equation

2.20 $$\begin{aligned} Ax^{m}\textrm{e}^{-ax}\big (1+g(x)\big )=z, \end{aligned}$$

and note that there exists a unique solution as long as *z* is sufficiently small. Then

2.21 $$\begin{aligned} x&=\frac{1}{a}\ln (1/z)+\frac{m}{a}\ln \big (\ln (1/z)\big )+\frac{\ln (A/a^m)}{a}+o(1), \quad z\rightarrow 0. \end{aligned}$$

#### Proof of Theorem 2.8

(i) Since the quantile function *Q*(*p*) solves the equation $F_Z(Q(p))=p$ (and recalling that $F_Z(-z)=\overline{F}_Z(z)$), by applying the limiting form (2.11) it can be seen that *Q*(*p*) solves an equation of the form (2.20) with x=ln(-Q(p)), and z=p, a=1, m=N-1 and $A=2^{(M+N-2)/2}\sigma _M/(\pi ^{(M+N)/2}(N-1)!s_N)$. By applying (2.21) with a=1, z=p, m=N-1 and x=ln(-Q(p)), we get that, as p→0,

2.22 $$\begin{aligned} Q(p)&=-\exp \Big (\ln (1/p)+\ln \big ((\ln (1/p))^{N-1}\big )+\ln (A)+o(1)\Big )\sim -\frac{A}{p}\big (\ln (1/p)\big )^{N-1}. \end{aligned}$$

The limiting form (2.12) now follows from inputting the above value of *A* into (2.22).

(ii) Upon applying the limiting form (2.15) to the equation $F_Z(Q(p))=p$ (and again recalling that $F_Z(-z)=\overline{F}_Z(z)$), it can be seen that *Q*(*p*) solves an equation of the form (2.20) with $x=(-Q(p))^{2/M}$, and $A=(2^{M/2-1}\sigma _M^{1/M})/\sqrt{M\pi }$, z=p, $a=M/(2\sigma _M^{2/M})$ and m=-1/2. By applying (2.21) with m=-1/2, z=p and $x=(-Q(p))^{2/M}$, we have, as p→0,

2.23 $$\begin{aligned} Q(p)&=-\bigg (\frac{1}{a}\ln (1/p)-\frac{1}{2a}\ln \big (\ln (1/p)\big )+\frac{\ln (Aa^{1/2})}{a}+o(1)\bigg )^{M/2} \sim -\bigg (\frac{1}{a}\ln (1/p)\bigg )^{M/2}, \end{aligned}$$

and the limiting form (2.13) follows by inputting $a=M/(2\sigma _M^{2/M})$ into (2.23). □

## Special cases

In this section, we present some special cases in which the general formulas for the PDF, CDF and characteristic function from Sect. 2 take a simpler form.

1. N=0: As mentioned in the introduction, in the case M=2, the PDF can be expressed in terms of the modified Bessel function of the second kind. In the M=2 case, a closed-form formula in terms of the modified Bessel function of the second kind and the modified Struve function of the first kind is also available; see (Gaunt 2022, Sect. 2.3). For M=2,3,4, simpler formulas, expressed in terms of elementary functions and Bessel functions, are available for the characteristic function; see (Gaunt and Li 2024, Remark 3.8).

2. M=0, N=1: the random variable Z=1/Y follows the reciprocal normal distribution with PDF $f_Z(z)=(\sqrt{2\pi }s_1z^2)^{-1}\exp (-1/(2s_1^2z^2))$, $z\in \mathbb {R}$. We can apply the reduction formula (A.38) to the general formula (2.6) for the characteristic function to obtain the following formula, which we could not find in the literature, for the characteristic function of Z=1/Y in terms of the $\phantom {a}_0F_2$ generalized hypergeometric function (see Olver et al. 2010, Chapter 16):

$$\begin{aligned} \varphi _Z(t)= \phantom {a}_0F_2\bigg (;\frac{1}{2},\frac{1}{2};\frac{t^2}{8s_1^2}\bigg )-\frac{\sqrt{\pi }|t|}{\sqrt{2}s_1}\,\phantom {a}_0F_2\bigg (;1,\frac{3}{2};\frac{t^2}{8s_1^2}\bigg ), \quad t\in \mathbb {R}. \end{aligned}$$

3. M=0, N=2: By the standard formula for the PDF of a one-to-one transformation of a random variable, we have $f_Z(z)=f_{1/Y}(z)=z^{-2}f_Y(1/z)$, $z\in \mathbb {R}$, and therefore

$$\begin{aligned} f_Z(z)=\frac{1}{\pi s_2z^2}K_0\bigg (\frac{1}{s_2|z|}\bigg ),\quad z\in \mathbb {R}. \end{aligned}$$

Also, since the distribution of *Z* is symmetric about the origin we have, for $z\in \mathbb {R}$,

$$\begin{aligned} F_Z(z)&= \frac{1}{2} +\frac{\textrm{sgn}(z)}{\pi s_2}\int _0^{|z|} \frac{1}{x^2}K_0\bigg (\frac{1}{s_2x}\bigg )\,\textrm{d}x= \frac{1}{2} +\frac{\textrm{sgn}(z)}{\pi }\int _0^{1/(s_2|z|)} K_0(u)\,\textrm{d}u\\&=\frac{1}{2}+\frac{1}{2s_2z}\bigg [K_0\bigg (\frac{1}{s_2|z|}\bigg )\textbf{L}_{-1}\bigg (\frac{1}{s_2|z|}\bigg )+\textbf{L}_0\bigg (\frac{1}{s_2|z|}\bigg )K_1\bigg (\frac{1}{s_2|z|}\bigg )\bigg ], \end{aligned}$$

where $\textbf{L}_\nu (x)$ is the modified Struve function of the first kind (Olver et al. 2010, Chapter 11). Here we evaluated the integral using (Gradshetyn and Ryzhik 2007, equation 6.561(4)) together with the basic identity $K_{-1}(x)=K_1(x)$ (see Olver et al. 2010, equation 10.27.3).

4. M=N: This case corresponds to the product of *M* independent Cauchy random variables. Applying the reduction formulas in (A.34) to the PDF formula (2.2) yields the following elementary formulas for the PDFs of $Z_2={X_1X_2}/({Y_1Y_2})$ and $Z_3={X_1X_2X_3}/({Y_1Y_2Y_3})$:

3.24 $$\begin{aligned} f_{Z_2}(z)&=\frac{2r_2\ln (r_2|z|)}{\pi ^2(r_2^2z^2-1)},\quad z\in \mathbb {R},\end{aligned}$$

3.25 $$\begin{aligned} f_{Z_3}(z)&=\frac{r_3(4\ln ^2(r_3|z|)+\pi ^2)}{2\pi ^3(r_3^2z^2+1)},\quad z\in \mathbb {R}, \end{aligned}$$

where $r_2=s_2/\sigma _2$ and $r_3=s_3/\sigma _3$. Formula (3.24) was earlier derived by Ride (1965) (in the case $\sigma _2=s_2=1$). Formula (3.25) was obtained by Springer and Thompson (1966) (in the case $\sigma _3=s_3=1$), who more generally gave an expression for the PDF of the product of *M* independent Cauchy random variables as a finite series expressed in terms of powers of ln(|z|), and gave closed-form formulas for M=1,2,…,10.

A closed-form formula, expressed in terms of the dilogarithm function, for the CDF of $Z_2$ can also be given (such a formula was not given in Ride (1965) or Springer and Thompson (1966)). The dilogarithm is a special case of the polylogarithm, which can be defined by the power series $\textrm{Li}_s(x)=\sum _{k=0}^\infty x^k/k^s$ for |x|<1 and by analytic continuation elsewhere; the dilogarithm $\textrm{Li}_2$ corresponds to the case s=2. Since the distribution of *Z* is symmetric about the origin, we have that $F_{Z_2}(z)=1/2+\textrm{sgn}(z)\int _0^{|z|} f_{Z_2}(x)\,\textrm{d}x$. Thus, for $z\in \mathbb {R}$,

3.26 $$\begin{aligned} F_{Z_2}(z)&=\frac{1}{2}+\frac{2\,\textrm{sgn}(z)}{\pi ^2}\int _0^{r_2|z|}\frac{\ln (x)}{x^2-1}\,\textrm{d}x=\frac{1}{2}+\frac{\textrm{sgn}(z)}{\pi ^2}\int _0^{r_2|z|}\bigg (\frac{\ln (x)}{x-1}-\frac{\ln (x)}{x+1}\bigg )\,\textrm{d}x\nonumber \\&=\frac{1}{2}+\frac{\textrm{sgn}(z)}{\pi ^2}\int _0^{r_2|z|}\bigg (\frac{\ln (x)}{x-1}+\frac{\ln (x+1)}{x}-\bigg (\frac{\ln (x)}{x+1}+\frac{\ln (x+1)}{x}\bigg )\bigg )\,\textrm{d}x\nonumber \\&=\frac{1}{2}+\frac{\textrm{sgn}(z)}{\pi ^2}\bigg [\frac{\pi ^2}{6}-\textrm{Li}_2(1-r_2|z|)-\textrm{Li}_2(-r_2|z|)-\ln (r_2|z|)\ln (1+r_2|z|)\bigg ], \end{aligned}$$

where we evaluated the integral using the integral formula $\int _1^x\ln (t)/(1-t) \,\textrm{d}t=\textrm{Li}_2(1-x)$ (see Olver et al. 2010, equation 25.12.2), the basic derivative $\frac{\textrm{d}}{\textrm{d}x}(\ln (x)\ln (x+1))=\log (x)/(x+1)+\log (x+1)/x$, and the specific values $\textrm{Li}_2(1)=\pi ^2/6$ and $\textrm{Li}_2(0)=0$. A closed-form formula, expressed in terms of the polylogarithm function, can also be given for the CDF of $Z_3$; however, the formula is rather complicated, so we do not report it.

Finally, we note that as a by-product of this analysis we obtain the following reduction formula for the Meijer *G*-function, which to the best of our knowledge is new.

### Corollary 3.1

Suppose $a\in \mathbb {R}$. Then, for x>0,

$$\begin{aligned} G_{3,3}^{2,3}\bigg (x \; \bigg | \;{\begin{array}{c} a+\frac{1}{2},a,a\\ \\ a,a,a-\frac{1}{2} \end{array}}\bigg )= 2x^{a-1/2}\bigg [\frac{\pi ^2}{6}-\textrm{Li}_2(1-\sqrt{x})-\textrm{Li}_2(-\sqrt{x})-\ln (\sqrt{x})\ln (1+\sqrt{x})\bigg ]. \end{aligned}$$

### Proof

Combine equations (2.4) and (3.26) and then apply the identity (A.30). □

5. M=2, N=1: Combining formulas (2.2) and (A.35), and formulas (2.5) and (A.36) yields the following formulas for the PDF and characteristic function of $Z=X_1X_2/Y_1$:

3.27 $$\begin{aligned} f_Z(z)&=\frac{s_1}{\sqrt{2}\pi ^{3/2}\sigma _2}\exp \bigg (\frac{s_1^2z^2}{2\sigma _2^2}\bigg )E_1\bigg (\frac{s_1^2z^2}{2\sigma _2^2}\bigg ), \quad z\in \mathbb {R}, \nonumber \\ \varphi _Z(t)&=\frac{1}{\sqrt{\pi }}\exp \bigg (\frac{\sigma _2^2t^2}{2s_1^2}\bigg )\Gamma \bigg (\frac{1}{2},\frac{\sigma _2^2t^2}{2s_1^2}\bigg ),\quad t\in \mathbb {R}, \end{aligned}$$

where $E_1(x)=\int _{0}^{\infty }\textrm{e}^{-t}/t\,\textrm{d}t$ is the exponential integral function and $\Gamma (a,x)=\int _x^\infty u^{a-1}\textrm{e}^{-u}\,\textrm{d}u$ is the upper incomplete gamma function.

6. M=1, N=2: From formula (3.27) we readily obtain the following formula for the PDF of $Z={X_1}/({Y_1Y_2})(=(Y_1Y_2/X_1)^{-1})$:

$$\begin{aligned} f_Z(z)=\frac{\sigma _1}{\sqrt{2}\pi ^{3/2}s_2z^2}\exp \bigg (\frac{\sigma _1^2}{2s_2^2z^2}\bigg )E_1\bigg (\frac{\sigma _1^2}{2s_2^2z^2}\bigg ),\quad z\in \mathbb {R}. \end{aligned}$$

7. M=3, N=1: In this case, we were unable to locate in the literature a reduction formula for the Meijer *G*-function that allows for a simpler formula to be given for the PDF. However, on applying the reduction formula (A.37) to (2.5) we obtain the following formula for the characteristic function of $Z=X_1X_2X_3/Y_1$:

$$\begin{aligned} \varphi _Z(t)= \frac{2\cos ^{-1}(\sigma _3|t|/s_1)}{\pi \sqrt{1-(\sigma _3|t|/s_1)^2}}, \quad t\in \mathbb {R}. \end{aligned}$$

## The Meijer *G*-function

In this appendix, we define the Meijer *G*-function, and state some of its relevant basic properties, which, unless otherwise stated, can be found in the standard references (Luke 1969; Olver et al. 2010).

The *Meijer G-function* is defined, for $x\in \mathbb {R}$, by the contour integral

A.28 $$\begin{aligned} G^{m,n}_{p,q}\bigg (x \, \bigg |\, {\begin{array}{c} a_1,\ldots , a_p \\ \\ b_1,\ldots ,b_q \end{array}} \bigg )=\frac{1}{2\pi \textrm{i}}\int _L\frac{\prod _{j=1}^m\Gamma (b_j-s)\prod _{j=1}^n\Gamma (1-a_j+s)}{\prod _{j=n+1}^p\Gamma (a_j-s)\prod _{j=m+1}^q\Gamma (1-b_j+s)}x^s\,\textrm{d}s, \end{aligned}$$

where the integration path *L* separates the poles of the factors $\Gamma (b_j-s)$ from those of the factors $\Gamma (1-a_j+s)$. We use the convention that the empty product is 1.

The *G*-function satisfies the identities

A.29 $$\begin{aligned} G_{p,q}^{m,n}\bigg (x \; \bigg | \;{\begin{array}{c} a_1,\ldots ,a_{p-1},b_1 \\ \\ b_1,\ldots ,b_q \end{array}}\bigg )&=G_{p-1,q-1}^{m-1,n}\bigg (x \; \bigg | \;{\begin{array}{c} a_1,\ldots ,a_{p-1} \\ \\ b_2,\ldots ,b_q \end{array}}\bigg ), \quad n>p;\, m,p,q\ge 1, \end{aligned}$$

A.30 $$\begin{aligned} x^\alpha G_{p,q}^{m,n}\bigg (x \; \bigg | \;{\begin{array}{c} a_1,\ldots ,a_p \\ \\ b_1,\ldots ,b_q \end{array}}\bigg )&=G_{p,q}^{m,n}\bigg (x \, \bigg | \,{\begin{array}{c} a_1+\alpha ,\ldots ,a_p+\alpha \\ \\ b_1+\alpha ,\ldots ,b_q+\alpha \end{array}}\bigg ),\end{aligned}$$

A.31 $$\begin{aligned} G_{p,q}^{m,n}\bigg (x \; \bigg | \;{\begin{array}{c} a_1,\ldots ,a_p \\ \\ b_1,\ldots ,b_q \end{array}}\bigg )&=G_{q,p}^{n,m}\bigg (x^{-1} \; \bigg | \;{\begin{array}{c} 1-b_1,\ldots ,1-b_q \\ \\ 1-a_1,\ldots ,1-a_p \end{array}}\bigg ). \end{aligned}$$

Combining equations 5.4(1) and 5.4(13) of Luke (1969), yields the following indefinite integral formula:

A.32 $$\begin{aligned} \int x^{\alpha -1}G_{p,q}^{m,n}\bigg (x \; \bigg | \;{\begin{array}{c} a_1,\ldots ,a_p \\ \\ b_1,\ldots ,b_q \end{array}}\bigg )\,\textrm{d}x=x^\alpha G_{p+1,q+1}^{m,n+1}\bigg (x \, \bigg | \,{\begin{array}{c} 1-\alpha ,a_1,\ldots ,a_p \\ \\ b_1,\ldots ,b_q,-\alpha \end{array}}\bigg ). \end{aligned}$$

The following limiting form is given in (Luke 1969, Sect. 5.7, Theorem 5): as $x\rightarrow \infty$,

A.33 $$\begin{aligned} G^{q,0}_{p,q}\bigg (x \; \bigg |\; {\begin{array}{c} a_1,\ldots , a_p \\ \\ b_1,\ldots ,b_q \end{array}} \bigg )\sim \frac{(2\pi )^{(\sigma -1)/2}}{\sqrt{\sigma }}x^\theta \exp \big (-\sigma x^{1/\sigma }\big ), \end{aligned}$$

where σ=q-p and $$\theta =\sigma ^{-1}\{(1-\sigma )/2+\sum _{i=1}^qb_i-\sum _{i=1}^pa_i\}$$.

We have the following reduction formulas:

A.34 $$\begin{aligned} G_{2,2}^{2,2}\bigg (x \; \bigg | \;{\begin{array}{c} 0,0\\ \\ 0,0 \end{array}}\bigg )&=\frac{\ln (x)}{x-1}, \quad G_{3,3}^{3,3}\bigg (x \; \bigg | \;{\begin{array}{c} 0,0,0\\ \\ 0,0,0 \end{array}}\bigg )=\frac{\ln ^2(x)+\pi ^2}{2(x+1)},\end{aligned}$$

A.35 $$\begin{aligned} G_{1,2}^{2,1}\bigg (x \; \bigg | \;{\begin{array}{c} 0\\ \\ 0,0 \end{array}}\bigg )&=\textrm{e}^xE_1(x),\end{aligned}$$

A.36 $$\begin{aligned} G_{1,2}^{2,1}\bigg (x \; \bigg | \;{\begin{array}{c} \frac{1}{2}\\ \\ 0,\frac{1}{2} \end{array}}\bigg )&=\sqrt{\pi }\textrm{e}^x\Gamma \bigg (\frac{1}{2},x\bigg ),\end{aligned}$$

A.37 $$\begin{aligned} G_{2,2}^{2,2}\bigg (x \; \bigg | \;{\begin{array}{c} \frac{1}{2},\frac{1}{2}\\ \\ 0,\frac{1}{2} \end{array}}\bigg )&=\sqrt{\frac{\pi }{x}}\Phi \bigg (1-\frac{1}{x},1,\frac{1}{2}\bigg ) =\frac{2\sqrt{\pi }\cos ^{-1}(\sqrt{x})}{\sqrt{1-x}},\end{aligned}$$

A.38 $$\begin{aligned} G_{0,3}^{2,0}\bigg (x \; \bigg | \;{\begin{array}{c} -\\ \\ 0,\frac{1}{2},\frac{1}{2} \end{array}}\bigg )&=\phantom {a}_0F_2\bigg (;\frac{1}{2},\frac{1}{2};x\bigg )-2\sqrt{\pi x}\,\phantom {a}_0F_2\bigg (;1,\frac{3}{2};x\bigg ), \end{aligned}$$

where these reduction formulas are suitable special cases of formulas that can be found at http://functions.wolfram.com/07.34.03.0887.01,http://functions.wolfram.com/07.34.03.1053.01,http://functions.wolfram.com/07.34.03.0724.01,http://functions.wolfram.com/07.34.03.0723.01,http://functions.wolfram.com/07.34.03.0880.01,http://functions.wolfram.com/07.34.03.0607.01, respectively. Here Φ(x,s,a) is the Lerch transcendent, which is defined by the power series $\Phi (x,s,a)=\sum _{k=0}^\infty x^k/(a+k)^s$ for |x|<1 and by analytic continuation elsewhere. We obtained the second equality in (A.37) by using the identities $\Phi (x,1,1/2)=2x^{-1/2}\tanh ^{-1}(\sqrt{x})$ and $\tanh ^{-1}(\sqrt{1-1/x})=-\textrm{i}\cos ^{-1}(\sqrt{x})$.
